# Phytotherapy in Alzheimer’s Disease—A Narrative Review

**DOI:** 10.3390/biomedicines12081812

**Published:** 2024-08-09

**Authors:** Julia Piekarz, Natalia Picheta, Oliwia Burdan, Marcelina Kurek, Magdalena Chrościńska-Krawczyk

**Affiliations:** 1 Students’ Scientific Association, Department of Paediatric Neurology, Medical University, 20-059 Lublin, Poland; natalia.picheta2812@gmail.com (N.P.); oliwia.burdan_in@interia.pl (O.B.); marcelinaqrek@wp.pl (M.K.); 2 Department of Children’s Neurology, University Children’s Hospital, 20-093 Lublin, Poland; magdalenachk@wp.pl

**Keywords:** Alzheimer’s disease, phytotherapy, *Curcuma*, *Berberis*, *Ginseng*, *Crocus*

## Abstract

Alzheimer’s disease (AD) affects 50–70% of patients with dementia, making it the leading cause of dementia. The condition is classified as a neurodegenerative, progressive and incurable disease. The disease is affecting more and more people around the world. AD has a multifactorial nature, spreading from beta-amyloid deposition to inflammation in patients’ brains. Patients experience cognitive impairment and functional decline. Although it is a disease that occurs mainly in the elderly, it is increasingly being diagnosed in young people between the ages of 30 and 40. It not only affects the patient themself but also reduces the quality of life of their closest caregivers. According to the WHO, the treatment of AD consumes USD 1.3 trillion globally, but it is only symptomatic, as there are no drugs to prevent the onset of AD or treat the cause of its onset. Due to the numerous side effects of therapy and the lack of proactive drugs that act on the pathomechanism of AD, alternative therapies are being sought. One possible option that has many studies confirming its effect is phytotherapy. Many herbs have pharmacological properties, such as antioxidant, anti-inflammatory, or neuroprotective effects, making them the future of cognitive disorders and AD treatment. This review focuses on some of the most promising herbs that have potentially potent properties and effects in AD therapy. These include *Curcuma longa*, *Panax ginseng*, *Berberis* and *Crocus sativus*. These herbs may perhaps be key in the future to make functioning and life easier for patients struggling with AD.

## 1. Introduction

Alzheimer’s disease (AD) is the leading cause of dementia worldwide. The disease is also becoming one of the most costly, deadly and intractable diseases of this century [1]. Considering the growing number of sufferers, it has been estimated that by 2050, the number of people with dementia worldwide will reach 152 million. The most vulnerable are low- and middle-income countries where access to doctors, expensive therapies and pharmaceuticals is limited. According to facts and accurate figures on this pathology from 2020, the number of AD patients over the age of 65 in America alone could increase significantly from 5.8 million to 13.8 million over the next 30 years [2]. Moreover, the number of deaths caused by the disease increased by 146.2% between 2000 and 2018, and AD became the fifth largest cause of death, making it one of the alarmingly growing diseases and rising statistics in meta-analyses on the epidemiology of the disease [3].

AD is a multifaceted disease with many hypotheses for the pathogenesis of the condition. One of them is oxidative stress. Excessive increases in ROS cause the initiation of apoptosis of nerve cells in the brain. In addition, aging and neuroinflammation further seeded the formation of oxidative stress. Another hypothesis is the stimulation of cholinergic neurotransmission in the brain by inhibiting the enzyme pathway [4]. Moreover, pathological processing of Aβ and tau protein is found. In addition, neurovascular changes and synaptic and mitochondrial dysfunctions also contribute to the potential onset and worsening of AD. Additionally, 5% of AD patients have a familial form of the disease. They are found to have mutations in the *presenilin 1* (*PSEN1*), *presenilin 2* (*PSEN2*), or the *Amyloid precursor protein (APP)* genes [5]. A number of disease mechanisms expand the potential points of entry for substances used in AD therapy [4].

When the symptoms of the disease are relatively mild, people can still participate in society, play sports, maintain good interactions with family, and even work. The patient’s condition deteriorates over time, there are difficulties in performing daily activities, and the patient may show aggressive behavior due to the lack of recognition of family members [6]. In the advanced stage of the disease, there may be problems with orientation and adjustment of the biological clock; the patient will constantly repeat one seemingly meaningful activity. Therefore, greater commitment, patience and intense vigilance of the patient’s family and caregivers are necessary [7]. In the final stages of the disease, damage extends to areas of the brain that prevent basic functions such as swallowing. At the end of life, patients are most often bedridden and require 24 h, full-time care, which often involves the family abandoning their current duties and social life in order to care for the patient [8].

AD is a disease that can cause various symptoms and wreak havoc on the patient’s body, which motivates the search for new methods of quick and accurate diagnosis and effective therapy. This is a pressing and important niche in medicine, as evidenced by statistics on morbidity and mortality and the fact that the median age at which AD is diagnosed is shifting towards an increasingly younger generation [9]. Taking into account the above-mentioned aspects, it is important to focus on therapeutic methods that may even prevent the development of the disease. Despite well-developed medicine and decades of clinical research into drugs for AD, the literature still only provides symptomatic treatment and not actual treatment of the cause. Donepezil, memantine, and galantamine are drugs that are currently the therapeutic gold standard and belong to two groups: anticholinesterase inhibitors and N-methyl-D-aspartate (NMDA) antagonists [10]. These therapies are administered orally or transdermally. They are not ideal solutions because they have a huge number of side effects and toxicity that force discontinuation of therapy [11]. The most troublesome side effects of the gold standard in the treatment and care process are insomnia, nightmares, muscle spasms, fatigue and anorexia [12]. The current state motivates the expansion of knowledge about the disease, its pathogenesis, symptoms and, most importantly, effective therapy. An innovative solution with great therapeutic success is the use of phytotherapy. This alternative method to previously known clinical approaches to managing AD offers great hope for patients and their families [13].

After an extensive analysis of herbs with potential indications in AD therapy, the herbs chosen were *Curcuma longa*, *Panax ginseng*, berberies and Crocus satibus. These herbs have the most studies confirming many pharmacological properties, such as anti-inflammatory, antioxidant, anti-apoptotic and antimitotic. Substances contained in the aforementioned herbs, after an extensive selection, were ranked among those most promising for AD drug therapy, so this narrative review is based on them. In addition, after reviewing articles on alternative AD therapies, it was found that there is still a lack of in-depth articles based on the pathomechanisms of the disease and the properties of herbs. This narrative review is based on the most recent research and has a very young bibliography. Expanding the knowledge of alternative therapy options is now essential for patients, as it gives them new hope, and many of them are increasingly seeking other therapeutic methods and are becoming convinced by phytotherapy. Therefore, this review provides a clear and readable way to acquaint readers with the latest research regarding the effectiveness and broad possibilities of AD phytotherapy.

## 2. Materials and Methods

The available literature was reviewed based on the Pubmed and Scopus databases. The keywords “Alzheimer’s disease”, “phytotherapy”, “*Curcuma*”, “*Ginseng*”, “*Berberis*” and “*Crocus*” were searched for 394,812 papers between 2012 and 2024. A total of 168,692 papers were found in Pubmed, while 226,120 papers were found in Scopus. A total of 126 articles were included in the narrative review, using the following inclusion criteria: original articles, reviews, book chapters and editorials, all of the above written in English only. Exclusion criteria included conference abstracts and duplicate articles. More than 95% of the included papers were published within the last 5 years.

## 3. Pathomechanism of Alzheimer’s Disease

Low levels of education, diabetes, inadequate diet, strokes, hypertension or physical activity are all risk factors that promote the development of the disease [14]. Understanding the pathomechanism of AD is still quite a challenge for researchers despite the fact that considerable progress has been made in describing and attempting to fill in the gaps in the pathogenesis of the disease. The most well-known and widespread is the beta-amyloid (Aβ) hypothesis, or senile plaques. They take different morphological forms and can be compact, diffuse or classic. Beta-amyloid is derived from the amyloid precursor protein (APP). It undergoes cleavage by β-secretases, resulting in non-toxic fragments, while when cleaved first by β-secretases and then by γ-secretases, neurotoxic beta-amyloid 42 is formed [15]. Aβ is mainly formed in endosomes, and its release from neurons is variable, depending on presynaptic and postsynaptic activity [16].

The Aβ hypothesis is combined with the inflammatory hypothesis, now recognized as the most significant element in pathogenesis. It is believed that Aβ stimulates the production of pro-inflammatory cytokines, which is related to amyloid’s structural similarity with small antimicrobial peptides, for example, cathelicidin, which provokes glial cells to produce pro-inflammatory cytokines. Additionally, amyloid can induce the formation of pores in cell membranes, which is also the domain of antimicrobial peptides that stimulate the immune system. Furthermore, as the brain ages, Aβ concentration increases, resulting in increased production of pro-inflammatory cytokines [17]. In addition, microglia are the main source of inflammatory factors produced in the brain. Although it tries to halt the progression of neurodegeneration in the early stages of the disease, it fails in later stages [18]. Both amyloid and tau protein accumulation can contribute to the activation of microglia. As a result of the presence of these two peptides, there is an increase in the proliferation and expression of inflammatory markers, i.e., CD36, CD14, CD11c, major histocompatibility complex II (MHC-II) and inducible nitric oxide (iNOS), as well as interleukin: Il-1 Il-6 and tumor necrosis factor (TNF), which stimulates microglia activation [19].

As for tau protein, it is a free transferrin that stabilizes nerve cells and is involved in axonal transport. When tau protein disconnects from microtubules and its hyperphosphorylation occurs, it becomes non-functional and begins to form neurofibrillary tangles (NFTs) inside neurons. Deposition of tangles causes synaptic dysfunction, leading to loss of synapses, which leads to neuronal degeneration. Initial aggregates of tau protein in the cytoplasm of neurons containing only single TP phosphoepitopes have also been identified, which are transversal lesions to tangles. The first site where tangles appear is the sinusoidal area, and later, the subcortical nuclei are involved [20]. The synergism of tau protein with Aβ is also increasingly reported; in addition, both exert a toxic effect on the vasculature, thereby leading to the breakdown of blood–brain barriers (BBB) [21].

There is increasing talk about the role of oxidative stress in brain aging and related AD. Reactive oxygen species (ROS) are metabolic products necessary for physiological function but can become toxic at high concentrations. Mitochondrial ROS are particularly dangerous, as mitochondrial DNA (mtDNA) lacks protection in the form of histones, making it more likely to mutate [22]. Mitophagy, the removal of damaged mitochondria, is then an important process. Damage to mtDNA is also associated with damage to nuclear DNA in the promoter region responsible for vesicle function, synaptic plasticity and mitochondrial function [23]. Poorly functioning mitochondria are one of the first markers of the developing disease, specifically an increase in mtDNA oxidation. It is found that the age-related loss of mitochondrial function affects APP processing and expression, resulting in the formation of beta-amyloid oligomers; at the same time, beta-amyloid is a source of ROS [22]. Additionally, the impairment of mitochondrial function in patients is further worsened due to impaired glucose metabolism and insulin production, and there is a decrease in the levels of peroxisome proliferator-activated receptor-γ coactivator 1α (PGC-1α), a transcription coactivator that regulates the expression of genes responsible for mitochondrial genesis, in the brains of AD patients [24].

**Figure 1:**
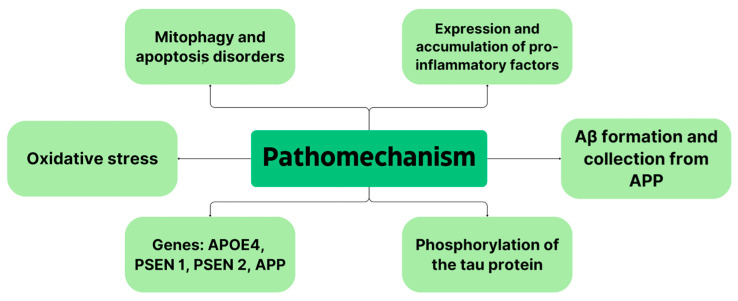
Summary of AD pathomechanism.

However, genetic predisposition is the strongest predisposing factor; 60–80% of AD risk is due to the presence of genetic factors. About 70 genes are involved in the pathogenesis of AD. The strongest of these is the apolipoprotein E (APOE) ε4 allele, a trigger for Aβ accumulation, and it is found in 40–65% of all AD patients [25]. Carrying one copy of this allele increases the risk of developing the disease by as much as three to seven times, while carrying two copies of this gene increases the risk by 12 times. In the brains of healthy individuals, APOE is expressed and secreted by astrocytes and microglia. It is then lipidated and internalized by APOE receptors like low-density lipoprotein receptor-related protein 1 (LRP1). This receptor is expressed in vascular smooth muscle cells, neurons, endothelial cells or pericytes. In turn, in the brains of AD patients, astrocytes and microglia respond to dense amyloid sheets, angiopathy-laden cerebral arteries and tangles, activating APOE transcription in microglia and reducing it in astrocytes while leading to altered lipid metabolism [26]. In contrast, APOE4 causes the stimulation of amyloid aggregation and deposition as amyloid plaques. It is speculated that APOE4 fragments cause neuronal dysfunction, astrogliosis and neurodegeneration and that N-terminal parts of APOE4 interact with mitochondria, decreasing their viability and impairing their function [25]. APOE4 increases Aβ accumulation by increasing APP processing by mitogen-activated protein kinase (MAPK), aggregating Aβ to oligomers and accelerating amyloid deposition in the brain [21]. Figure 1 summarizes the most important aspects of AD pathomechanism.

## 4. Herbs

### 4.1. Curcuma longa L.

Turmeric is a plant-based curry spice that originated in India and belongs to the Zingiberaceae family. It is currently used in many other countries of the World, such as Latin America, China and Asia. The global market for turmeric is ~1.7 million tons. While the value of the global market in 2016 is set at USD 0.5 billion [27].

The compound is considered safe for daily use at a dose of 0–3 mg/kg by the U.S. Food and Drug Administration (FDA), the FAO/WHO Joint Expert Committee on Food Additives (JECFA) and the European Food Safety Authority (EFSA) [28].

Turmeric contains turmeric oil and curcuminoids: curcumin (77%), demethoxycurcumin (17%) and bis-demethoxycurcumin (3%). The main polyphenolic compound of turmeric rhizome, which is curcumin, has the greatest therapeutic properties [29]. The compound has many pharmacological activities: anti-inflammatory, anti-amyloidogenic, anti-diabetic, anti-aging, antioxidant or antimicrobial [30]. In addition, it is found to have beneficial effects on the following gastrointestinal problems: diarrhea, indigestion, vomiting and flatulence. It is also found to have medicinal potential in AD, Parkinson’s disease, cardiovascular disease and chronic obstructive pulmonary disease [31]. It has beneficial effects on acetyl esterase inhibition, tau suppression and copper binding and enhances Aβ phagocytosis by microglia [32]. Curcumin passes through the blood–brain barrier, but due to its poor solubility and low bioavailability after oral administration, its effect is limited [30]. Over the past few years, researchers have searched for many methods that would improve the solubility and bioavailability of curcumin due to its broad and promising pharmacological activities. Numerous formulations of nanocurcumin have been produced so that the permeability of the substance has improved significantly [33]. In addition, the bioavailability of curcumin was found to increase when it was administered simultaneously with the herb Ginkgo biloba [29].

### 4.2. Panax ginseng

Ginseng root is a remedy that has been used for more than 2000 years in East Asian countries, such as Korea, China and Japan, and contains many beneficial actions on the human body [34,35]. The countries of China, Canada, the United States and South Korea are responsible for 99% of its production [36]. In 2012, the herb was approved by the Chinese government as a foodstuff and raw material in medical products [37]. Global demand for the herb at the end of 2016 was 5.2 million kilograms, and the global ginseng market is projected to be worth USD 7.51 billion by 2025 [38].

The plant belongs to the Araliaceae family of the genus Panax [39]. It is called the “queen of all herbs” due to its numerous pharmacological properties: anti-inflammatory, anti-allergic, hypoglycemic, antioxidant, anti-inflammatory, memory-enhancing, anti-aging, cardioprotective, neuroprotective, anticancer [39,40,41]. In addition, its effects on improving immune function, blood pressure and human metabolism are noted [39].

Compounds found in Ginseng include ginsenosides, phytosterols, sesquiterpenes, flavonoids, polyacetylenes, alkaloids, amino acids or phenolic compounds. It contains many substances, which is why its use in medicine is so widespread [39]. Ginsenosides (TGS) are divided into two groups: protopanaxadiol type (PPD) and protopanaxatriol type (PPT). The former includes nine compounds, with Rc, Rd, Rb2, Rb1, Rb3, m-ginsenoside Rb1, m-ginsenoside Rc, m-ginsenoside Rb2, m-ginsenoside Rd. On the other hand, the second includes six substances: Re, Rg1, Rf, 20(R)-ginsenoside Rg2, notoginsenoside R1, m-ginsenoside Re [37]. The aforementioned compounds have potential wide applications in neurology, diabetology, or oncology [39]. Phenolic compounds, such as elemicin and dauricine, have anti-inflammatory, antioxidant and anti-inflammatory effects [38].

### 4.3. Berberis L.

Plants of the *Berberis* genus contain berberine (an isoquinoline alkaloid), which has been widely used for a long time in traditional medicine. There are 17 genera and as many as 650 species of the plant worldwide [42]. Among the well-known are *B. vulgaris*, *B. jaeschkeana*, B. *aristata*, *B. integerrima*, *B. dasystachya* and *B. koreana* [43]. These plants are widely grown in Europe, the United States, South Asia, Iran or Pakistan. It is used in the food, tea, confectionery and pharmaceutical industries [44].

*Berberis* extract contains many active substances. In addition to berberine, which is rich in pharmacological properties, tetrandrin, chondrocurin, palmatine, flavonoids (chrysantamine, hyperoside, dolfinidin-3-O-beta-D-glucoside, pelargonin, petunidin-3-O-beta-D-glucoside, alpha-tocopherol and beta-carotene) also occur [45].

The active alkaloid has many pharmacological properties: antimicrobial, antidiabetic, anticancer, antioxidant or anti-inflammatory, antidiabetic, antidyslipidemic [46]. In addition, it has been found to have neuroprotective effects, so it has potential broad applications in neurology [47].

### 4.4. Crocus sativus

*Crocus sativus* is commonly known as saffron and otherwise known as “red gold”. The largest producers of the plant from the Iridaceae family are Iran, Greece, Morocco, Spain and India [48]. Since ancient times, it has been used as a spice, perfume, dye and traditional medicine [49]. It was used in ancient Rome as an additive to wine to prevent poisoning. In Egypt and Greece, it was used to combat ulcers on the skin and mucous membranes to treat urinary disorders and eye diseases [50].

The main active substances found in saffron are the carotenoids that give the plant its color: crocin and crocetin. Apocarotenoids, such as picrocrocin, impart flavor, and terpenes, such as safranal, impart an aroma. In addition, there are polyphenols and flavonoids [48]. Saffron contains about 30% crocin, 5–15% picrocrocin and 2.5% safranal [51].

Saffron has many pharmacological properties: analgesic, anti-inflammatory, hepatoprotective, sleep-inducing, antioxidant, antimicrobial, antidiabetic, antinausea, antidepressant, antispasmodic, antianxiety, anticonvulsant, antitussive and neuroprotective [51,52,53].

### 4.5. Herbal Summary

**Figure 2:**
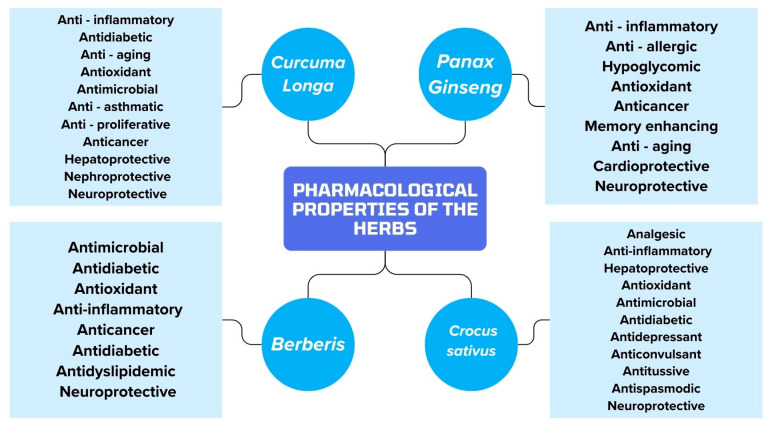
Summary of the pharmacological properties of the herbs included in the article.

Figure 2 shows the pharmacological properties of *Curcuma longa*, *Panax ginseng*, *Berberis* and *Crocus sativus*.

## 5. Alzheimer’s Disease Therapy

Therapy of the disease is based on inhibiting the progression and reversal of symptoms [54]. Many drugs are used but none of them act directly on the pathology of the disease. Two groups of drugs are mainly used in AD therapy: acetylcholinesterase enzyme inhibitors and NMDA antagonists [55].

The first drug introduced from the AChEI group is tacrine, but due to its hepatotoxicity, it is no longer currently used [56]. Donepezil, rivastigmine and galantamine are the three AChEI drugs currently used for the symptomatic treatment of AD [57]. Inhibition of the enzymes acetylcholinesterase (AChE) and butyrylcholinesterase (BChE) increases acetylcholine (ACh) concentrations in the brain and may contribute to the blockade of Aβ plaque formation [56]. All of these drugs manifest adverse symptoms: dizziness, agitation, falls, diarrhea, confusion and emotional problems [58]. In addition, they can also cause bradycardia, heart block, rhabdomyolysis or neuroleptic malignant syndrome [9].

Memantine is an antagonist of the NMDA or ionotropic glutamate receptor. They are primarily involved in synaptic plasticity underlying learning and memory. In addition, they are counted among the main actors in excitotoxic damage occurring during chronic neurodegenerative injury [59]. The most common side effects include dizziness, confusion, diarrhea or constipation. In addition, neurological abnormalities can often be seen, such as abnormal gait, seizures, drowsiness, late dyskinesia and malignant neuroleptic syndrome. The drug can also affect heart rate, liver function and kidney function [11].

In June 2021, the FDA approved aducanumab, a human monoclonal antibody (IgG1) that selectively targets aggregated soluble and insoluble forms of Aβ [60,61]. In AD patients, it causes a dose- and time-dependent reduction in brain β-amyloid levels. Patients were reported to experience dizziness, edema, microbleeds, diarrhea, falls, disorientation and impaired consciousness after using the drug [60]. In May 2023, the FDA approved brexpiprazole for the treatment of agitation associated with AD-induced dementia, and in July 2023, lecanemab, which is an antibody with high affinity for soluble forms of Aβ, was also approved [62,63,64]. All of the drugs currently approved and used to treat AD affect only the inhibition of the symptoms of the disease. Currently, there is no drug available on the market that affects the pathogenesis of the disease. In addition, each drug has many adverse effects. Due to these drawbacks of AD therapy, potential other therapeutic methods are being sought. One of them is phototherapy, which can provide AD patients with many benefits [65].

**Figure 3:**
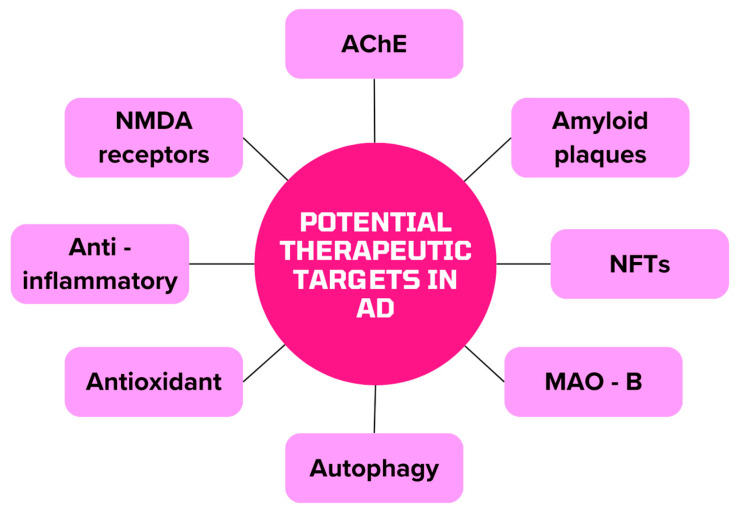
Potential therapeutic targets for AD alternative therapy (MAO-B monoamine oxidase B).

Figure 3 shows the potential capture points of the active substances contained in herbs and drugs that can be used to treat AD.

## 6. Alternative Therapies for Alzheimer’s Disease

### 6.1. Curcuma longa

Curcumin or 5-hydroxy-1,7-bis(4-hydroxy-3-methoxyphenyl)-1,4,6-heptatrien-3-one) is a phenolic compound from the rhizome of *Curcuma longa*, which is found in 3–5% in the plant [66,67]. Curcumin supplementation has a neuroprotective effect [68]. It induces neurogenesis within the hippocampus and protects dopaminergic neurons [66,68]. This occurs through the activation of nuclear erythroid factor 2 (Nrf2), an increase in β-tubulin, neurogenin, neuroligin, neuroD1 and neuregulin [66].

The role of oxidative stress in AD pathology has been increasingly mentioned. It is stated that curcumin is categorized as a peroxisome proliferator-activated receptor γ (PPARγ) agonist, resulting in improved mitochondrial function and reduced ROS formation. In addition, it has been noted that the compound has the effect of inhibiting the activation of microglia by lipopolysaccharides, thus reducing the production of nitric oxide (NO) and the pro-inflammatory cytokines IL-6 and IL-1β [69]. Curcumin is responsible for inhibiting lipid peroxidation, by which amyloid accumulation and oxidative stress-induced neurotoxicity are reduced [70].

Curcumin is also credited with inhibiting presenilin-1 (PS-1), which is responsible for the production of Aβ from APP, consequently potentially reducing further Aβ synthesis [70]. In addition, curcumin has broad anti-inflammatory properties, as it inhibits cytokine synthesis by down-regulating cyclooxygenase-2 (COX–2), iNOS, TNF-α, IL-1, IL-2, IL-6, IL-8 and IL-12 [71].

Curcumin is responsible for inhibiting dysregulated signaling pathways in cells [72]. Insulin, together with insulin-like growth factor (IGF-1), is responsible for regulating tau protein phosphorylation. A decrease in their concentration is responsible for hyperphosphorylation of the protein and loss of neurons in the hippocampus [66]. Additionally, the phosphatidylinositol 3-kinase/protein kinase B/glycogen synthase kinase-3β (PI3K/Akt/GSK-3β) pathway is involved in the development of AD. Neurotrophins, such as nerve growth factor (NGF) and brain-derived neurotrophic factor (BDNF), are responsible for the activation of the pathway, while the accumulation of Aβ is responsible for the down-regulation and over-activation of GSK-3β [73]. Over-activated kinase causes an increase in Aβ synthesis, the accumulation of amyloid plaques, impairs synaptic plasticity and increases neuronal degeneration and cognitive impairment in patients [74]. Curcumin leads to the inhibition of GSK-3β, thus slowing the development and pathology of AD [66].

The study included 29 patients between the ages of 30 and 70. Fifteen of them were assigned to the control group, which took two placebo tablets. The study group included 14 people taking two tablets of curcumin (Meriva) in a dose of 500 mg. After 12 weeks, GSK-3β and islet amyloid polypeptide (IAPP) levels were examined in both groups. The results showed a significant decrease in the concentration of both substances in the study group. The absolute change in serum GSK-3β in those taking curcumin was −2.4 ± 0.4 ng/mL, and IAPP was −2.0 ± 0.7 ng/mL. In contrast, in the group taking placebo, the absolute change was analogously −0.35 ± 0.05 ng/mL and 0.4 ± 0.05 ng/mL [75].

A study was conducted on three patients with dementia who were given 100 mg/d of curcumin. It was noted that cognitive function improved in 33.3% of the subjects. After 12 weeks of taking the substance, the number of points on the Mini-Mental State Examination (MMSE) increased from 12 to 17 [76].

In a study on a transgenic mouse model of AD, curcumin was administered at a dose of 160 ppm, and it was inferred that there was a reduction in total insoluble amyloid burden and amyloid plaques by 39% and 43%, respectively. In addition, it was concluded that there was a 61.8% reduction in IL-1β cytokine expression [30].

The study used 30 rats, which were divided into three groups. The control group had 10 animals receiving distilled water. In the second group, streptozotocin (STZ) was administered to induce a model of AD in the rats, followed by distilled water. The last group, on the other hand, contained animals with the AD disease model, which were administered curcumin nanoparticles (CN) at a dose of 50 mg/kg. After 15 days, they showed a significant decrease in tau immunoreactivity in the group receiving CN; this was 10% in the cortex and −2% in the hippocampus. At the same time, in the group of rats with the AD model, the values were analogously 50% and 28%. In addition, acetylcholinesterase (AChE) activity was examined. The decrease in the activity of the enzyme in rats receiving CN indicates the inhibition of acetylcholine (ACh) breakdown [77]. Table 1 shows the changes in AChE activity in the brain and hippocampus before and after CN use, indicating significant inhibition of the enzyme after CN use.

The effects of curcumin (CUR) on the development and course of AD and *ginkgo biloba* (GBE) on improving CUR permeability across the BBB were studied. The study used male rats that were induced to develop cognitive impairment based on a modified model of AD by the intraperitoneal administration of scopolamine 4 mg/kg and an oral mixture of heavy metals (HMM). Animals were administered memantine (MEM) at a dose of 20 mg/kg, CUR 100 mg/kg and CUR + GBE 100 mg/kg + 400 mg/kg. An immunohistochemical analysis of Aβ and tau in rat cells was performed, and a significant decrease in proteins was inferred in MEM-, CUR- and CUR + GBE-treated rats compared to the AD model control group (SCO + HMM). The concentration of Aβ protein in SCO + HMM was 7.833 ± 0.4773, where it analogously decreased to 3.667 ± 0.3333, 3.333 ± 0.2108, 0.3333 ± 0.2108 after using MEM, CUR, CUR + GBE. On the other hand, adequately, the concentration of tau protein in the control group taking only saline and carboxymethylcellulose (CMC) was 5500 ± 0.500, and after using the same substances, it analogously decreased to 2333 ± 0.3333; 2167 ± 0.1667 and 0.1667 ± 0.1667. In addition, Table 2 shows the positive effect of the combination of CUR + GBE so that CUR can reach much higher concentrations in plasma and hippocampus, which will greatly improve the therapeutic effect of CUR in AD therapy. The present results indicate the positive potential of CUR for AD patients [29].

**Figure 4:**
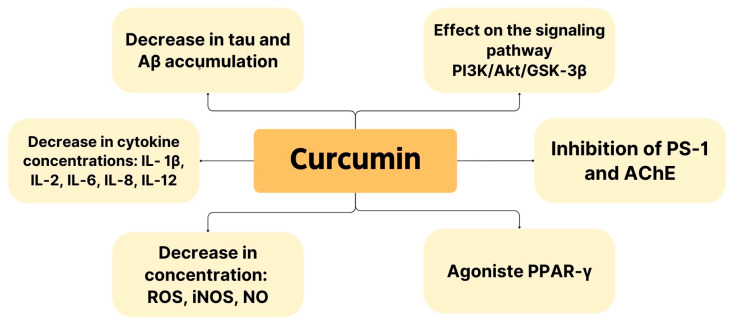
Pharmacological properties showing potential use of curcumin in AD therapy.

Figure 4 shows the pharmacological properties of curcumin, as demonstrated in the studies included in this narrative review. These properties demonstrate the potential for *Curcuma longa* to be used in AD therapy.

### 6.2. Panax ginseng

*Ginseng* is an herb whose neuroprotective effects are mainly based on counteracting AD pathomechanism cascades, Aβ formation, anti-apoptosis, anti-inflammatory effects and mitochondrial dysfunction [78]. Ginsenosides are among the most promising substances found in *Ginseng* [36].

Rg1 is a compound with potential use in AD. It enhances microglia function, consequently reducing Aβ deposits. In addition, correlations of the substance with the enhancement of mitophagy, which removes abnormal or damaged mitochondria, are noted. This occurs via the serine/threonine protein kinase1-E3 ubiquitin ligase (PINK1-PARKIN) pathway. In dysfunctional mitochondria, PINK1 binds to the outer mitochondrial membrane (OMM) and regulates the translocation of PARKIN, which sequesters damaged mitochondria [79,80]. The disruption of PINK1-PARKIN results in the accumulation of damaged mitochondria, which will consequently disrupt cell and brain bioenergetics. This will lead to ATP deficiency, impaired cell signaling and cellular dysfunction and/or brain death [81]. In addition, Rg1 is found to improve hippocampal function by increasing the proliferation of its cells and consequently improving memory. Increased mRNA expression and production of glial cell-derived neurotrophic factor (GDNF), BDNF and NGF have also been noted [82]. Rg1 is a substance that is well degraded by intestinal bacteria after oral administration, so an alternative route for its administration is parenteral or intranasal. It was found that after nasal administration, the transport time to the brain was reduced by 100%. In contrast, the distribution and transport efficiency of Rg1 increased by 5.05 and 2.5 times [83].

Rb1 is another substance contained in *Ginseng* that has beneficial applications in AD. Its neuroprotective effects are based on its anti-apoptotic effect, increasing the number of neurons and stimulating the expression of kinases responsible for this pathway [83]. It stimulates Schwann cell proliferation and NGF and BDNF expression [78].

Rd, Rb2, and Rg3 are among the substances with potent AChE inhibitory effects, so they can have a positive effect in patients struggling with cognitive and memory impairment [84]. In addition, they inhibit the expression of PS-1 and β-amyloid cleaving enzyme 1 (BACE-1), through which Aβ production is inhibited [78].

Rd is responsible for reducing neuronal apoptosis due to the inhibition of the apoptosis signal-regulating kinase 1/c-Jun N-terminal kinases (ASK1-JNK) pathway [36]. ASK1 activation occurs via ROS and intracellular calcium overload, which results in the initiation of cell apoptosis. On the other hand, JNK is responsible for regulating the process via anti-apoptotic (Bcl-2, Bcl-xl, Bcl-w) and pro-apoptotic (Bad, Bax, Bak) proteins [85]. In addition, there is an increase in the number of hippocampal dentate gyrus cells through the signaling pathway PI3K/Akt [36]. The former pathway is responsible for regulating survival, proliferation, increased differentiation, cell motility and neurite elongation. PI3K is activated by BDNF, so an increase in this factor has a beneficial effect on AD inhibition. It is concluded that the described pathway has an important function in the matter of maintaining the adequate synaptic plasticity of brain cells. In addition, it is noted that they play an important function in the consolidation of long-term memory in the hippocampus. AKT, on the other hand, is responsible for regulating proteins involved in the signaling pathway [86].

Re, through activation of PPAR γ, causes the inhibition of BACE-1, resulting in the inhibition of Aβ production. In addition, choline acetyltransferase (ChAT) and vesicular acetylcholine transporter (VAChT) expression was found to be upregulated, which was associated with an increase in cellular Ach levels and was beneficial in inhibiting AD progression [40].

The study involved 66 SAMP8 mice and 12 SAMR1 mice, which were administered donepezil 1.6 mg/kg, Rb1 (30 or 60 µmol/kg) or Rg1 (30 or 60 µmol/kg). Aβ protein levels in the hippocampus were analyzed, and it was concluded that Rb1 and Rg1 could be used in AD therapy. The relative density of Aβ/β-actin in SAMP8 mice was 0.7. However, after treatment with Rb1 (30 µmol/kg), Rb1 (60 µmol/kg), Rg1 (30 µmol/kg), Rg1 (60 µmol/kg) adequately decreased to 0.4, 0.35, 0.6, 0.3. Thus, it can be inferred that these substances significantly reduced the concentration of Aβ protein [87].

Another study evaluated the change in tau concentration in the olfactory bulb, spinal cord and brain after Rd application. APP transgenic mice aged 10 months were used. They were given intraperitoneal injections of Rd 10 mg/kg once a day for 6 months [88]. Table 3 shows the relative intensity of tau proteins in the brain, spinal cord and olfactory bulb before and after Rd application.

A study was performed to determine the efficacy of Rg1 in AD. A 5XFAD mouse model was used, and 10 mg/kg/d of Rg1 was administered intraperitoneally before 30 days. In addition, human SH-SY5Y cells induced by β-amyloid oligomer (AβO) or APPswe overexpression were treated with 1 μM Rg1. Based on the results, serine-threonine protein kinase (mTOR) and unc-51-like kinase 1 (ULK1) phosphorylation was inhibited, making mitophagy more efficient [79]. The mTOR protein is responsible for the proper functioning of nerve cells by regulating mitochondrial function, protein synthesis, energy metabolism and autophagy. Additionally, it is found to be responsible for axon regeneration and formation [89]. In contrast, ULK1 is responsible for the activation of autophagy [85,90]. Furthermore, increased PINK1- Parkin signaling and more efficient mitochondrial degradation were found. APP expression was found to be reduced in three experimental models. It was found that the size of deposits decreased by more than 50% [79].

The study included 40 AD patients aged 50 to 90 years. They were divided into four groups differing in the dose of SG-135 [a capsule containing ginsenosides: Rb1 (4.5%), Rb2 (4.8%), Rc (4.9%), Rg3 (23.8%), Rk1 (12.3%) and Rg5 (13.1%)] of 1.5 g/d, 3 g/d, 4.5 g/d and a control group. The analysis of the results was based on the evaluation of the scores obtained in the tests: Alzheimer’s Disease Assessment Scale—Cognitive Subscale (ADAS-cog), ADAS-non-cog and MMSE. It was found that after 12 and 24 weeks of therapy, the higher the dose of SG-135, the greater the increase in MMSE scores and the decrease in ADAS scores, which proved the beneficial effect of ginsenosides on improving cognitive function in AD patients [91].

**Figure 5:**
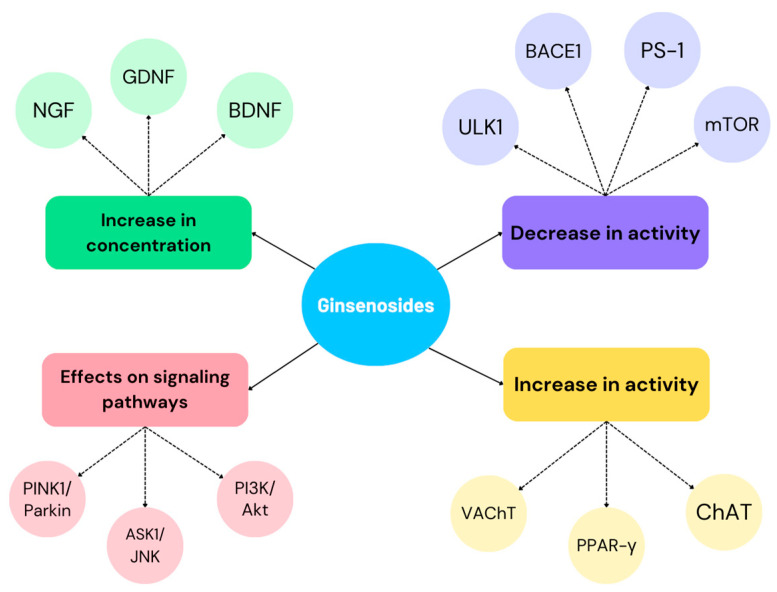
Pharmacological properties of ginsenosides affecting the inhibition of AD progression and symptoms.

Figure 5 shows the pharmacological properties demonstrated in the studies included in this narrative review. These point to the potential use of ginsenosides in the inhibition of AD symptoms and therapy.

### 6.3. Berberis

*Berberis* contains berberine, which has many medicinal properties. As early as the 1970s, its effects on the nervous system were described. Initial studies showed its sedative effects, but its therapeutic activity has been expanded to include ischemic disease, AD, depression, anxiety, epilepsy and Parkinson’s disease [92]. The bioavailability of berberine after oral administration is limited. After intravenous administration, the substance penetrates the blood–brain barrier better, so it reaches significant concentrations in the hippocampus. In addition, it then has better and faster metabolism and clearance [93].

Berberine has the ability to reduce the formation of extracellular amyloid plaques and intracellular neurofibrillary tangles [94]. It reduces the expression of BACE1 by activating the extracellular signal-regulated kinases 1/2 (ERK 1/2) pathway so that Aβ levels are reduced [95]. The ERK 1/2 is responsible for regulating the growth, apoptosis and differentiation of neural cells. Platelet-derived growth factor (PDGF), NGF and epidermal growth factor (EGF) are responsible for activating the signaling pathway [96]. In addition, berberine has been noted to affect the protein kinase RNA-like endoplasmic reticulum kinase/eukaryotic translation initiation factor-2α (PERK/eIF2α) signaling pathway. PERK activation is stress-dependent and is responsible for an increase in eIF2α phosphorylation, which results in an increase in BACE1 activity. As a consequence, Aβ production and accumulation are inhibited, and neuronal apoptosis occurs [97]. The level of tau protein significantly decreases by strongly reducing its hyperphosphorylation [95].

Berberine can increase dopamine, norepinephrine and serotonin levels in brain cells. In addition, it decreases the activity of diphosphohydrolase of ectonucleoside triphosphate (NTPDase) and 5′-nucleotidase [98]. In addition, it affects the inhibition of AChE, butyrylcholinesterase (BChE) and monoamine oxidase (MAO) [99].

Berberine’s antioxidant capacity is important in inhibiting the disease or its symptoms [99]. In this study, we evaluated the antioxidant properties of berberine and a substance whose berberine phenolic groups were detached, resulting in the polyphenolic Ber-D. The antioxidant properties were compared with vitamin C (Asc). The results showed that Ber-D at a dose of 50 µM almost matched the percentage antioxidant capacity (%AC) of the vitamin (95% versus 100%) [100].

The study testing the efficacy of berberine used 3xTg AD mice. It was randomly divided into a control group (12 mice) and a research group (12 mice), which was administered 100 mg/kg/d of berberine for 4 months. Analysis of total tau protein concentration showed that the control group had a relative protein expression of 1.0, while the research group had a relative protein expression of 0.6. The activities of the enzymes GSK3β, protein phosphatase 2 (PP2A) and Akt, which are responsible for tau dephosphorylation, were also examined. Their activity was examined by a ratio that inversely reflected their activity. GSK3β and PP2A are responsible for as much as more than 70% of tau phosphatase activity in the brain. The results shown in Table 4 indicate that berberine decreased GSK3β and Akt activity while it increased PP2A, which had a beneficial effect on reducing tau phosphorylation. [101].

The test substance causes an increase in Akt activity. In addition, the study analyzed the effect of berberine on the protein sequestosome 1 (P62/SQSTM1) and cathepsin D levels. Both substances are involved in the autophagy–lysosomal pathway. The former is involved in directing ubiquitinated proteins to autophagic vacuoles. The second, on the other hand, is a major aspartyl protease, the concentration of which is high in the brain. It is activated by proteolysis in acidified lysosomes, and the mature product m-cathepsin D is formed. Berberine caused a decrease in relative protein expression to 0.6 (control group 1.0). In contrast, it increased cathepsin D to 1.1 (control group 1.0) and m-cathepsin D to 1.3 (control group 1.0) [101].

The study used 30 APP/PS1 transgenic mice, which were divided into three groups of 10 animals each. The control group received a lower dose of berberine (BBR) of 50 mg/kg and a higher dose of BBR of 100 mg/kg. The effect of berberine on oxidative stress was analyzed. The activities of several enzymes involved in neutralizing oxidative stress were examined. Glutathione peroxidase (GPx-1/2) is responsible for the antioxidant defense of the nervous system by scavenging free radicals. Glutathione synthetase (GSS) is responsible for increasing the concentration of glutathione in the brain, which is responsible for the antioxidant defense of tissues. Glutathione reductase (GR) reduces glutathione. The activity of all the enzymes of these enzymes increased significantly in mice taking berberine [102].

The anti-inflammatory properties of berberine were also investigated. The researchers used astrocyte AND microglia biomarkers to detect anti-inflammatory properties. Anti-GFAP (glial fibrillary acidic protein) was used to assess the number of biomarkers in astrocytes, while anti-CD 45 was used for glial. In addition, inflammatory markers were measured: IL-1β: interleukin 1 beta; TNF-α: tumor necrosis factor alpha. The results shown in Table 5 indicate that berberine has anti-inflammatory and antioxidant properties. It decreases the concentration of CD45, GFAP, TNF-α and IL-1β. At the same time, it increases the content of GPx-1/2, GSS or GR. These results indicate that the substance has potential applications in the treatment of Alzheimer’s disease [102].

The study used 36 3xTg- AD mice, which were divided into three groups of 12 animals each. The groups differed in ingestion: 50 mg/kg/d berberine, 100 mg/kg/d berberine and drinking water (control group). The properties of berberine to increase autophagy in hippocampal neurons were analyzed. The increase in autophagy was probably due to the induction of the PI3K/Beclin-1 class III pathway [103]. The proteins tested were microtubule-associated protein light chain 3-II (LC3-II), P62, Bcl-2, Cathepsin-D and Beclin-1. LC3-II is responsible for binding to isolated membranes and producing autophagosomes. Together with the P62 protein, it performs autophagosome degradation [104]. Bcl-2 is counted as a mediator of autophagy inhibition, and its lower concentration results in an increase in LC3-II expression [105]. Beclin-1 is involved in the autophagy process from the memento of autophagosome formation to its maturation and elongation. It cooperates with PI3K, making the process more efficient [106]. On the other hand, cathepsin-D is an essential protease responsible for regulating the proteolytic activity of lysosomes. In situations of protein deficiency, there is an accumulation of proteins found in neurodegenerative pathologies [107]. Analysis of the results showed that there was a decrease in the expression of P62 and Bcl-2 but an increase in LC3-II, Cathepsin-d and Beclin-1. These changes confirm that berberine enhances autophagy in neurons and could potentially have an indication in AD therapy [103]. Table 6 shows the expression level of enzymes involved in autophagy. The results clearly indicate the properties that berberine enhances this process.

The study used 20 female 3xTg AD mice, half of which were administered berberine at a dose of 50 mg/kg/d. In addition, 10 wild-type C57 mice were used as a control group. Analysis of the results showed that berberine inhibits neuronal damage in the hippocampus and improves memory. Using staining and assessing the size, density and distribution of neurons, the occurrence of neuronal damage can be assessed. Hippocampal neurons in the control group were dense, properly organized and had full and distinct nuclei. In contrast, in the AD model group, the cells were discolored, deformed and loosely arranged. In the AD model mice treated with berberine, the neurons resembled those in the control group. Neuronal counts in the control group were 95, 75 in the AD model, and 90 in the berberine-treated mice [108].

**Figure 6:**
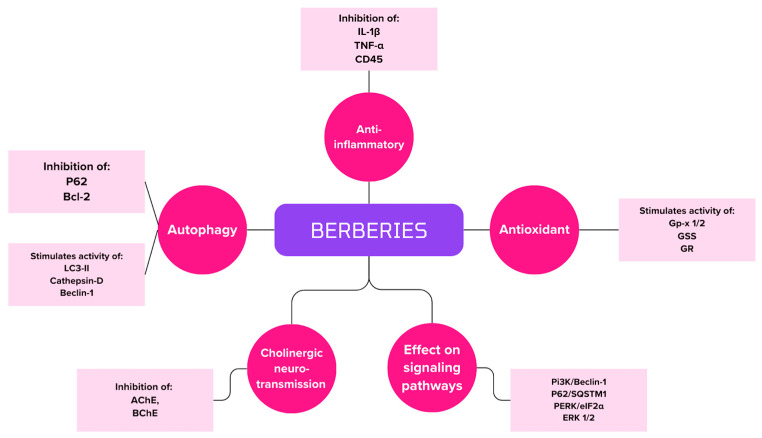
Summary of berberine handle points in AD therapy.

Figure 6 summarizes berberine’s grip points for therapy and the inhibition of AD symptoms.

### 6.4. Crocus sativus

*Crocus sativus*, commonly known as saffron, contains crocin, crocetin and saffronal, which have neuroprotective effects and could potentially be used in patients for AD therapy [109]. All substances reduce the expression of proteins Bad, Bax and caspase-3, by which apoptosis is inhibited and neuronal viability is increased [110]. They increase the expression of mTOR and IL-10 levels and decrease IL-1β and IL-6 [111]. In addition, they are attributed to the inhibition of GSK3β and ERK1/2 kinase and the inhibition of tau phosphorylation [112]. Saffron extract exerts antioxidant effects by decreasing NO release and ROS production and increasing glutathione (GSH), which neutralizes free radicals [110]. In addition, it reduces the activity of the antioxidant enzymes superoxide dismutase (SOD) and GPx-1/2 [113]. It also affects the decrease in AChE activity [114]. In addition, it inhibits tau aggregation and NFT production [115].

Crocetin is a potential inducer of autophagy, as it is mediated by serine/threonine kinase 11 (STK11/LKB1) to activate the AMP-activated protein kinase (AMPK) pathway. As a consequence, Aβ clearance is increased. In addition, it inhibits Aβ production by inhibiting BACE1 and PS-1 and increasing the ability of monocytes to degrade the protein. Additionally, it down-regulates GSK-3β and Mapk1/ERK2 [116].

The study used rats, which were divided into six groups. The first was a control group receiving saline. The rest of the animals were injected with Aβ peptide 0.5 µL on each side of the CA1 area of the hippocampus and given one of the substances, saline or crocin, at doses of 150 nmol/side, 300 nmol/side, 600 nmol/side via injection or 30 mg/kg intraperitoneally. During the study, the levels of Beclin-1 and caspase-3 and the ratio of LC3-II/LC3-I and Bax/Bcl-2 were analyzed. An increase in Beclin-1 activity, along with the LC3-II/LC3-I ratio, is responsible for an increase in the autophagy process in cells. In contrast, a decrease in caspase-3 activity results in a decrease in apoptosis. In addition, a decrease in the Bax/Bcl-2 ratio reports inhibition of apoptosis. Based on the analysis of the results shown in Table 7, it was deduced that crocin possesses anti-apoptotic properties and enhances autophagy [117].

In a study conducted on 46 patients, the valence of saffron for improving cognitive function was evaluated. In a double-blind study, patients were divided into two groups: the first taking saffron 30 mg/d and the second with a placebo. The results of the ADAS-cog and Clinical Dementia Rating Scale (CDR) tests showed that after 16 weeks of therapy, patients taking the herb experienced significant improvements in cognitive function. In addition, the substance appeared to be safe for use in the treatment of mild/moderate AD. Subsequently, the study was expanded, in which 54 AD patients were given either 30 mg/d of saffron or 10 mg/d of donepezil. The efficacy of therapy with both substances was similar, but significantly fewer side effects were noted during therapy with the herb [118].

The study used 30 ICR mice, which were divided into three groups: control (sham), Aβ 25–35 injected groups and Aβ 25–35 + crocin 40 mg/kg/d injected groups. Analysis of the results showed that crocin inhibited damage to hippocampal neurons in the hematoxylin and eosin stain (H&E) test. It was noted that in Aβ rats, cells had an incomplete structure and were sparsely distributed. After the application of crocin, the cells were approximated, and their structures were less affected. In addition, the anti-inflammatory properties of crocin were tested by IL-1β, IL-6 and TNF-α levels. The results showed that the relative expression of IL-6/βactin and IL-1 β/βactin in the control group was 1.0, the Aβ group was 1.6 and those using crocin were 0.7. Similarly, the relative expression of TNF-α/βactin was 1.0, 1.5, 1.2. In addition, the phosphorylation of PI3K and Akt was analyzed. Analysis of the results showed that there was inhibition in the Aβ model, while those receiving crocin were activated. The relative expression of p-PI3K/PI3k in the control group was 1.0, the Aβ model was 0.75, and during crocetin intake, it was 1.1. Similarly, the relative expression of p-Akt/Akt was 1.0, 0.6 and 1.1 [119].

In a study evaluating the active ingredient in *Crocus sativus* that is safranal, 66 Wistar rats were used. The animals were divided into six groups: a sham group (Sham), a sham group receiving safranal 0.2 mL/kg, and an Aβ group and an Aβ group receiving safranal at doses of 0.025 mL/kg, 0.1 mL/kg or 0.2 mL/kg. Substances responsible for oxidative stress, inflammation or apoptosis were evaluated. Table number 9 shows changes in the concentrations, or activity, of the compounds after safranal treatment. The results shown in Table 8 are a decrease in ROS, Il-1β, IL-6, TNF-α, caspase-3, AChE and an increase in catalase, SOD and GSH activity. These indicate the anti-inflammatory and antioxidant properties of safranal, which can be used in AD therapy [120].

The double-blind, randomized trial involved 68 patients with moderate to severe AD. Half of the patients took 20 mg/d of memantine, while the other half took 30 mg/d of saffron extract. Patients were assessed before and after therapy using the MMSE and Severe Cognitive Impairment Rating Scale (SCIRS). Sixty patients completed the study, 30 in each group. Analysis of the results showed that there were no significant differences in test scores between patients taking memantine and saffron. In the group taking the herbal extract, the mean (SD) difference between the initial and final values (after 12 months) of the SCIRS test was 1.88, and the MMSE was 1.29. Similarly, in the memantine-treated group, it was 1.61 and 1.67 [121].

**Figure 7:**
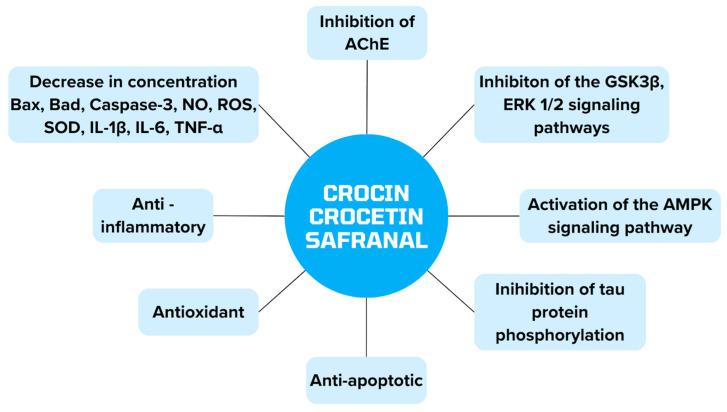
Presentation of the properties of substances contained in *Crocus sativus* in alternative therapy of AD.

Figure 7 summarizes the mechanisms of action of crocin, crocetin and safranal, which are contained in *Crocus sativus* and can be used in alternative AD therapy.

## 7. Summary

Table 9 summarizes the preclinical and clinical studies included in the narrative review using AD therapy herbs.

## 8. Discussion

AD is a disease that is increasingly being diagnosed around the world. It is one of the serious, difficult and burdensome conditions not only for the patient but also for their loved ones. Disease progression and increasingly advanced symptoms are common in these patients. The causes of the onset of AD are manifold and include genetic predisposition, environmental predisposition and older age [6].

AD therapy is a challenge for modern medicine due to the incomplete understanding of the pathogenesis of the disease and the lack of drugs to effectively halt the progression of mild AD [54]. Doctors should present and propose all possible treatments so that the patient has the right to decide and be fully informed about possible therapies for his condition.

The etiopathogenesis of the disease is incredibly broad, which allows for more potential points of resolution of a therapeutic substance, especially alternative therapies [55]. Unfortunately, current AD treatment is based solely on inhibiting the onset and progression of symptoms. Unfortunately, it is associated with numerous side effects, prompting patients to seek other alternative therapies, often reaching precisely for phytotherapy.

Phytotherapy has been used in traditional medicine for many years and is very popular and effective. According to the World Health Organization (WHO), about 85% of the population of developing countries use plants and their products in healthcare [122]. It is used in adjunctive therapy or monotherapy to treat urinary tract infections, diabetes, gastrointestinal problems or chronic pain [123,124]. The FDA has classified many of the herbs as generally recognized as safe (GRAS). *Curcuma, Ginseng* and *Crocus* have been classified as such [125].

The medicinal substances from the plants included in this narrative review have many promising mechanisms that could be used in AD therapy. There have been many advanced animal studies testing the efficacy of phytotherapy in treating AD. The results of most studies have been very promising. Studies have also been performed on human patients with AD. In those cases, significant improvements in cognitive function were noted, with fewer side effects occurring. Although there are quite a few studies confirming the efficacy and safety of phytotherapy in the treatment of AD, this pool should be expanded. It is worth expanding it with long-term studies and identifying active phytochemicals during the production of basic herbal products [126].

Selected after careful selection, the herbs described in the review have many pharmacological properties. *Curcuma longa* contains curcumin, which has anti-inflammatory, antioxidant and anti-apoptotic properties. It reduces Aβ production and tau protein phosphorylation. In addition, it reduces the concentration of ROS, iNOs, NO, IL-1β and IL-6, and AChE activity. It also affects the PI3K/Akt/GSK-3B pathway. *Panax ginseng* contains a variety of ginsenosides and also has many valuable properties. It affects an increase in VAChT, PPAR- γ, ChAT activity but decreases BACE1, PS-1 and mTOR. In addition, it affects the following signaling pathways: PINK1/Parkin, ASK1/JNK and Pi3K/Akt. *Berberies* is widely used in many branches of medicine. Its antioxidant, anti-inflammatory and anti-apoptotic properties can be used in AD therapy. By inhibiting AChE and BChE, they improve cholinergic transmission in the brain. They will reduce Bcl-2, IL-1β and TNF- α levels but increase GSS and GR activity. In addition, they will affect the following signaling pathways: PI3K/Beclin-1, P62/SQSTM1 and ERK 1/2. The last herb included in the review is *Crocus sativus*, which has several promising substances: crocin, crocetin and safranal. All of these compounds work by inhibiting tau protein phosphorylation and inhibiting AChE. In addition, they affect the following various signaling pathways: GSK-3B, ERK 1/2 or AMPK. They also reduce the concentration of many substances responsible for inflammatory, oxidative or apoptotic effects. These include Bax, Bcl-2, caspase-3, IL-1β or IL-6. All of these plants contain numerous substances rich in many pharmacological properties that can be used in alternative AD therapy.

Nowadays, many people use phytotherapy, take numerous dietary supplements and seek alternative therapies. They believe that natural treatment will have a much better effect on their health than pharmacologically produced drugs. Therefore, it is worth informing patients about the possibility of using herbs as an additional therapeutic source. Substances of plant origin currently mainly support the treatment of diseases, including AD, but perhaps soon, the future of the treatment of many problematic diseases, including AD, will be phytotherapy.

## 9. Conclusions

AD therapy is a heavy branch of medicine. Because of this, alternative therapeutic methods are being sought to curb the symptoms or to act as a causal therapy. Phytotherapy is a promising branch that could augment therapeutic methods for this condition. It has many chemical-rich substances that have numerous pharmacological actions, which could make it easier for patients to function. However, efforts should be made to expand the knowledge of herbs, and many long-term and human studies should be performed to fully confirm the effectiveness of this alternative therapeutic method. In addition, thanks to the widely developing field of medicine and the growing interest of researchers, medics and patients, perhaps in the future, AD therapy will be mainly based on supportive or causal therapy just using phytotherapy.

## Figures and Tables

**Table 1:** AChE activity in cortex and hippocampus [77].

Activity μmol SH/min/g	Control	Model AD	Model AD
Cortex	1.1	2.7	1.9
Hippocampus	1.7	3.6	1.5

**Table 2:** Effect of GBE on plasma CUR absorption and CUR distribution in the hippocampus at 30 and 60 min [29].

CUR Concentration in:	Plasma (ng/mL)	Hippocampus (ng/g)
CUR after 30 min	57.31 ± 4.134	49.46 ± 3.763
CUR + GBE after 30 min	80.58 ± 3.297	175.9 ± 8.346
CUR after 60 min	60.38 ± 2.747	119.7 ± 3.069
CUR + GBE after 60 min	100.3 ± 5.463	409.5 ± 6.766

**Table 3:** Relative intensity of tau protein in brain, spinal cord and olfactory bulb before and after Rd 10 mg/kg intake in mice [88].

Relative Intensity Tau Proteins	Brain	Spinal Cord	Olfactory Bulb
Baseline	5–7	4–7	3–7
Rd 10 mg/kg post-application value	1–3	1–2	1–7

**Table 4:** Concentration of enzymes involved in tau hyperphosphorylation [101].

Relative Protein Expression	p-GSK3β/GSK3β	p-Akt/Akt	p-PP2A/PP2A
Control group	1.0	1.0	1.0
Research group	1.3	1.35	0.8

**Table 5:** Relative density (OD) of substances evaluated after berberine treatment to determine their antioxidant and anti-inflammatory properties [102].

Relative OD (% of β-actin)	Control Group	Berberine 50 mg/kg/d	Berberine 100 mg/kg/d
GPx-1/2	0.55	1.55	1.7
GSS	0.55	1.6	1.65
GR	0.65	1.8	1.7
CD45	0.8	0.25	0.3
GFAP	1.25	0.5	0.45
IL-1β	1.4	0.6	0.65
TNF-α	1.3	0.6	0.5

**Table 6:** Change in expression of proteins involved in autophagy after berberine treatment at different doses [103].

Expression Level	LC3-II	P62	Bcl-2	Cathepsin-D	Beclin-1
Control	0.9	1.25	1.2	0.8	0.75
Berberine 50 mg/kg/d	1.1	0.9	0.9	1.0	1.1
Berberine 100 mg/kg/d	1.4	0.65	0.75	1.05	1.3

**Table 7:** Results of changes in the concentrations of proteins involved in apoptosis and autophagy after treatment with crocin [117].

Arbitrary Unit	Caspase-3	Beclin-1	Bax/Bcl-2	LC3-II/LC3-I
Control	1.0	1.0	1.0	1.0
Aβ	2.8	1.4	1.3	1.3
Aβ + Crocin 150 nmol/side	0.8	1.5	0.8	1.4
Aβ + Crocin 300 nmol/side	0.6	1.7	0.7	1.5
Aβ + Crocin 600 nmol/side	0.55	1.8	0.6	1.6

**Table 8:** Results of changes in the concentrations of proteins involved in apoptosis, anti-inflammatory and autophagy after treatment with safranal [120].

Activity/Concentration Level	Sham	Sham + Safranal 0.2 mL/kg	Aβ	Aβ + Safranal 0.025 mL/kg	Aβ + Safranal 0.1 mL/kg	Aβ + Safranal 0.2 mL/kg
ROS (AFU)	80	75	140	135	100	90
Catalase (Unit/mg)	2	2.2	1	1.2	1.4	1.6
SOD (Unit/mg)	4	4.2	2	2.4	3	3.2
GSH (nmol/mg)	4	3.9	2.6	2.8	3.4	3.6
IL-1β (pg/mg)	25	28	45	40	38	28
IL-6 (pg/mg)	22	24	42	40	30	28
TNF α (pg/mg)	28	26	44	42	34	32
Caspase-3 (OD)	0.6	0.7	1.2	1.1	0.9	0.8
AChE (nmol/min/mg)	28	30	48	42	36	38

**Table 9:** Studies included in a narrative review.

Year	Plant	Substance	Dose	Population	Result	Citation
2024	*Crocus sativus*	Crocin	40 mg/kg/d	30 ICR mice	Decrease in expression of IL-1β, IL-6, TNF- α Increase in PI3K and Akt activity	[115]
2023	*Curcuma longa*	Curcumin	160 ppm	Transgenic mice	Decrease in IL-1β expression	[29]
2023	*Curcuma longa*	Curcumin	100 mg/kg	48 rats	Decrease in tau and Aβ protein levels	[28]
2023	*Panax ginseng*	Rg1	10 mg/kg/d 1 uM	5XFAD mice	Inhibition of mTOR and ULK1 Increased PINK-Parkin pathway and autophagy	[74]
2023	*Berberis*	Berberine	50 mg/kg/d	Human SH-SY5Y cells	Inhibition of neuronal damage Improving memory	[104]
2022	*Curcuma longa*	Curcumin	50 mg/kg	20 mice 3xTg AD 10 wild type C57 mice	Decrease in AChE activity and tau protein levels	[72]
2021	*Panax ginseng*	Rd1	10 mg/kg	30 rats	Decrease in the concentration of tau protein	[83]
2021	*Crocus sativus*	Crocin	30 mg/d	APP transgenic mice	Improving cognitive functions	[114]
2020	*Curcuma longa*	Curcumin	500 mg	29 people	Decrease in GSK3β activation and IAPP levels	[70]
2020	*Panax ginseng*	Rb1 Rg1	30/60 µmol/kg 30/60 µmol/kg	66 SAMP8 mice 12 SAMR1 mice	Decrease in the concentration of Aβ protein	[82]
2020	*Berberis*	Berberine	100 mg/kg/d	24 mice 3xTg AD	Increase in PP2A and Akt activity Increase in autophagy Decrease in GSK3β activity	[97]
2019	*Panax ginseng*	Ginsenosides	1 g/d 3 g/d 4.5 g/d	40 people	Improved cognitive function in tests: MMSE, ADAS	[86]
2019	*Crocus sativus*	Safranal	0.025 mL/kg 0.1 mL/kg 0.2 mL/kg	66 Wistar rats	Increase in concentration/activity of GSH, catalase, GSH, SOD Decrease in concentration/activity of ROS, IL-1β, IL-6, TNF, Caspase-3 and AChE	[116]
2017	*Berberis*	Berberine	50 mg/kg 100 mg/kg	30 APP/PS1 mice	Increased activity of antioxidant enzymes: GPx-1/2, GSS, GR Decrease in inflammatory markers	[98]
2017	*Berberis*	Berberine	50 mg/kg/d 100 mg/kg/d	36 mice 3xTg AD	Increase in activity of LC3-II, Cathepsin-D, Beclin-1 Decrease in the activity of P62 and Bcl-2	[99]
2016	*Crocus sativus*	Crocin	150 nmol/side 300 nmol/side 600 nmol/side 30 mg/kg	Mice	Decrease in Caspase-3 activity Increase in Beclin-1 and LC3-II activity	[113]
2014	*Crocus sativus*	Saffron extract	30 mg/d	68 people	No significant difference in improvement of MMSE and SCIRS scores compared with memantine treated group	[117]

## Data Availability

Not applicable.

## Abbreviations

**Abbreviation**	**Meaning of the Abbreviation**
AD	Alzheimer’s disease
WHO	World Health Organization
NMDA	N-methyl-D-aspartate
Aβ	Beta-amyloid
APP	Amyloid precursor protein
PSEN 1	*Presenilin 1*
PSEN 2	*Presenilin 2*
MHC-II	Major histocompatibility complex II
iNOS	Inducible nitric oxide
TNF	Tumor necrosis factor
NFTs	Neurofibrillary tangles
BBB	Blood–brain barriers
ROS	Reactive oxygen species
mtDNA	Mitochondrial DNA
PGC-1α	Peroxisome proliferator-activated receptor-γ coactivator 1 α
APOE	Apolipoprotein E
LRP 1	Lipoprotein receptor-related Protein 1
MAPK	Mitogen-activated protein kinase
FDA	Food and Drug Administration
JECFA	FAO/WHO Joint Expert Committee on Food Additives
EFSA	European Food Safety Authority
TGS	Ginsenosides
PPD	Protopanaxadiol type
PPT	Protopanaxatriol type
Nrf2	Nuclear erythroid factor 2
PPAR γ	Peroxisome proliferator-activated receptor γ
NO	Nitric oxide
PS-1	Presenilin-1
IGF-1 AChEI	Insulin-like growth factor Acetylcholinesterase inhibitor
COX-2	Cyclooxygenase-2
NGF	Nerve growth factor
BDNF	Brain-derived neurotrophic factor
PI3K	Phosphatidylinositol 3-kinase
Akt	Protein kinase B
GSK-3β	Glycogen synthase kinase-3β
IAPP	Islet amyloid polypeptide
MMSE	Mini-Mental State Examination
STZ	Streptozotocin
CN	Curcumin nanoparticles
AChE	Acetylcholinesterase
ACh	Acetylcholine
CUR	Curcumin
GBE	Ginkgo biloba
HMM	Heavy metal
SCO + HMM	Model control group
MEM	Memantine
CMC	Carboxymethylcellulose
OMM	outer mitochondrial membrane
PINK1-PARKIN	Serine/threonine protein kinase1-E3 ubiquitin ligase
GDNF	Glial cell-derived neurotrophic factor
BACE-1	β-amyloid cleaving enzyme 1
ASK1-JNK	Apoptosis signal-regulating kinase 1/c-Jun N-terminal kinases
ChAT	Choline acetyltransferase
VAChT	Vesicular acetylcholine transporter
mTOR	Serine-threonine protein kinase
ULK1	Unc-51-like kinase 1
AβO	β-amyloid oligomer
ADAS-cog	Alzheimer’s Disease Assessment Scale—Cognitive Subscale
ERK 1/2	Extracellular signal-regulated kinases 1/2
EGF	Epidermal growth factor
PERK	Protein kinase RNA-like endoplasmic reticulum kinase
eIF2α	Eukaryotic translation initiation factor-2α
BChE	Butyrylcholinesterase
MAO	Monoamine oxidase
NTPDase	Ectonucleoside triphosphate
Asc	Vitamin C
%AC	Antioxidant capacity
PP2A	Protein phosphatase 2
P62/SQSTM1	Protein sequestosome 1
GSS	Glutathione synthetase
GPx-1/2	Glutathione peroxidase
GR	Glutathione reductase
GFAP	Glial fibrillary acidic protein
OD	Relative density
LC3-II	Microtubule-associated protein light chain 3-II
GSH	Glutathione
SOD	Superoxide dismutase
STK11/LKB1	Serine/threonine kinase 11
AMPK	AMP-activated protein kinase
CDR	Clinical Dementia Rating Scale
H&E	Hematoxylin and eosin
SCIRS	Severe Cognitive Impairment Rating Scale
SD	Mean
